# Associated Factors of Male Participation in Antenatal Care in Muaro Jambi District, Indonesia

**DOI:** 10.1155/2022/6842278

**Published:** 2022-05-18

**Authors:** Guspianto, Ismi Nurwaqiah Ibnu, Al Asyary

**Affiliations:** ^1^Study Program of Public Health Science, Faculty of Medicine and Health Sciences, Universitas Jambi, Muaro Jambi, Indonesia; ^2^Faculty of Public Health, Universitas Indonesia, Depok, Indonesia

## Abstract

**Objective:**

This study aims to evaluate the level of male participation and factors associated with male participation in antenatal care.

**Methods:**

A cross-sectional study was performed, involving a survey of 381 men, selected through multistage random sampling. The outcome variable male participation in antenatal care was constructed from eight dichotomized indicators, and measurement results were low (scored 1 and 2) and high (scored 3 and 4). Multiple logistic regression analysis was performed using SPSS 24.0 at a significance level of 0.05.

**Results:**

The percentage of male participation in antenatal care was low (41.2%). Associated factors included age (OR = 1.858, 95%CI = 1.066–3.240), number of children (OR = 2.909, 95%CI = 1.532–5.522), income (OR = 1.715, 95%CI = 1.060–2.775), and knowledge (OR = 3.706, 95%CI = 2.320–5.919). Knowledge was found to be the main factor for male participation in antenatal care in Muaro Jambi Regency.

**Conclusion:**

Male participation in antenatal care in Muaro Jambi District was low and was influenced by age, number of children, income, and knowledge. Health promotion programs are needed to empower men to participate in antenatal care by providing communication, education, and information.

## 1. Introduction

Increasing male participation that emphasizes responsibility in maternal health is the mandate of the International Conference on Population and Development (ICPD), which was held in Cairo in 1994 [1]. Studies have reported the benefits of male involvement in maternal health, such as increased access to antenatal care, which increases deliveries with trained personnel [2], increases access to the use of modern contraceptive methods [3], and overcomes barriers in accessing maternal health services [4]. Moreover, male participation promotes gender equality in families [5]. One form of male participation in maternal health involves playing an active role during the wife's antenatal care (ANC).

The involvement of men can delay decision making to take advantage of beneficial ANC health services. Maternal mortality is a major problem in various countries, including Indonesia. Maternal mortality rate (MMR) is a barometer of maternal health services and an indicator of a country's public health status [6]. The World Health Organization estimates that approximately 830 women die each day due to pregnancy and childbirth. MMR in Indonesia was 305 per 100,000 live births in 2016, which is the second highest in Southeast Asia after Laos [7]. In Jambi Province, MMR increased in the last 3 years, namely, 53 per 100,000 live births (2015), 56 per 100,000 live births (2016), and 59 per 100,000 live births (2017) [8]. Therefore, efforts to reduce MMR is a priority for Sustainable Development Goals, with a target of 70 per 100,000 live births in 2030 [9].

Male participation in ANC increases utilization of maternal health services [10–13]. Although the importance of male participation in ANC is being recognized, it remains minimal, especially in developing countries [14]. Low male participation rate in ANC was reported to reduce efforts in improving health and experience obstacles, resulting in maternal mortality [15, 16]. The low rate of male participation in ANC is because maternity care is considered the woman's concern and men are not ready to participate in ANC. Additionally, the ANC program prohibits male participation [17]. The low participation of men in ANC can lead to increased maternal morbidity and mortality due to delays in making decisions to get ANC services.

Factors reportedly associated with male participation in ANC include occupation, ethnicity, religion, waiting time, staff information, and attitude [17, 18]. A study reported that men's participation in reproductive health services is significantly related to education, employment, income, access to media, and number of children alive [19]. Green et al. (1991) suggested that male participation in ANC is a form of health behavior that is influenced by three factors, namely, predisposing factors as antecedent factors in behavior; supporting factors that enable the behavior to be performed; and drivers to reinforce behavior change [20]. This study aims to assess the level of male participation and analyze factors associated with male participation in ANC.

## 2. Materials and Methods

### 2.1. Design and Sampling

This was a quantitative study, with cross-sectional design. Data collection was performed from June to August 2020. The study was conducted in the Muaro Jambi area, a district in Jambi Province, covering an area of 5,246 km^2^, with a population of 365,700 people in 2019. This regency is located around the city of Jambi, which is the capital of Jambi Province. The region has three hospitals and 22 public health centers, which provide ANC services. This region was selected based on data on the low coverage of maternal services in government health-care facilities in Jambi Province.

The study sample included men of reproductive age, who lived with their partners and have had biological children aged up to 2 years at the time of data collection. The minimum sample size of 308 respondents was calculated based on Slovin's formula [21], with an error tolerance level of 5%, in addition to 10% of anticipated nonresponders, such that total samples included 438 respondents. Sample selection used a multistage sampling technique. Four subdistricts were randomly selected from 11 subdistricts; then, two villages were randomly selected from each subdistrict to obtain eight villages. Furthermore, the sample was randomly selected based on a household survey in eight proportionally selected villages.

### 2.2. Data Management and Analysis

Data collection was performed using a questionnaire developed by adapting from several indicators of the maternity planning and complications prevention program. This questionnaire was managed for internal structure validity (>0.3; in all questions using the item-total correlation) as well as with Cronbach's alpha (>0.60) to determine instrument reliability. The informed consent form was included on the first page of the questionnaire, and ethical approval was obtained from the health research ethics commission of the Health Polytechnic of the Ministry of Health Jambi, No: LB.02.06/2/109/2020.

The dependent variable—male participation in ANC—was constructed as a composite variable of eight questions with dichotomous answers (yes or no), including (1) planning the time and place for ANC with the wife; (2) taking the wife for pregnancy test; (3) accompanying the wife during pregnancy examinations; (4) discussing wife's pregnancy with health workers; (5) helping pregnant wife with household chores; (6) reminding the wife to exercise during pregnancy; (7) not smoking near pregnant wife; and (8) preparing potential blood donors for emergencies during pregnancy. Total score was calculated by adding scores of all activities the respondent reported. The scoring data are shown as continuous variables, which have nonnormal distribution. Participation level was categorized into low or high based on the cutoff (median) value. The median, as the threshold of the variable status, is allowed when no conventional standard or cutoff has been set beforehand, whether internationally or nationally (e.g., in assessing indoor air quality) [22].

The independent variable comprised 17 characteristics and background variables of respondents based on Green's theory (1991) [20]. Knowledge and service quality are composite variables of several question items categorized based on the cutoff point (median) value.

Univariate analysis was conducted to obtain the frequency and percentage of respondent characteristics and backgrounds as well as the level of male participation in ANC. The chi-square test was used to evaluate the relationship between independent and dependent variables and identify factors that associate with level of male participation in ANC using multiple logistic regression.

## 3. Results

### 3.1. Respondent Characteristics and Backgrounds

This study has a response rate of 87%. Of 438 participants, 381 were interviewed. Attempts to meet the remaining (13%) were unsuccessful after repeated home visits (Table 1).

### 3.2. Level of Male Participation in ANC

Our findings revealed that the highest proportion of male participation in ANC was planning the time and place for ANC with wives (66.4%) and the lowest was preparing potential blood donors for pregnancy-related emergencies (28.6%). This finding indicates that most respondents (58.5%) have low participation in ANC (Table 2).

### 3.3. Factors Related to Male Participation in ANC

Chi-square analysis of the relationship between respondent characteristics and background and the level of male participation in ANC revealed that 9 of 17 variables were significantly related to male participation, namely, age, number of children, income, husband's education, wife's education, knowledge, communication, social media information, and culture (Table 3).

### 3.4. Associated Factors of Male Participation in ANC

Results from multivariate analysis (logistic regression) revealed four factors associated with level of male participation in ANC, namely, age, number of children, income, and knowledge. Men aged >30 years tended to have a higher participation rate in ANC than men aged ≤30 years (OR = 1.858, 95%CI = 1.066–3,240). Men with ≤2 children tended to participate in ANC more than men with >2 children (OR = 2.909, 95%CI = 1.532–5.522). Men with higher incomes were more likely to participate in ANC than those with lower incomes (OR = 1.715, 95%CI = 1.060–2.775). Men with more knowledge tended to have higher participation in ANC than those with less knowledge (OR = 3.706, 95%CI = 2.320–5.919). Knowledge is the main factor correlating with level of male participation in ANC (Table 4).

## 4. Discussion

The role of men in maternal health has been described in detail in the literature. In developing countries, such as Indonesia, this role is especially important because of the culture of paternalism, which tends to favor males and discourages women from decision making [23, 24]. The findings of this study indicate that the level of male participation in ANC is low (41.2%). These are consistent with findings that male participation in ANC is low in many regions of the world, such as sub-Saharan Africa, Kenya, Ethiopia, and Uganda [13], and inconsistent with findings from Tanzania, Rwanda, Nigeria, England, and Uganda [2].

The SIAGA (*Siap Antar Jaga*) program is an effort to prevent the negative impact of pregnancy. Here, the husband is ready to act and take care of his pregnant wife or face childbirth as a form of participation or support. We found several variables that simultaneously have a significant relationship with male participation in the care of pregnant women, namely age, number of children, income, and knowledge. This finding further adds to the complexity of the problem of men's involvement in maternal health, especially ANC.

Support and participation of men as husbands on standby are important during pregnancy [25]. A study in Indonesia based on data from the 2012 Indonesian Demographic and Health Survey found that husbands tended to accompany their wives during pregnancy and childbirth examination when the age of wives ranged from 21 to 35 years (that is, the husbands were older) [26]. The findings of this study emphasize upon the importance of raising the legal age of marriage by both the government and society, which correlates with men's involvement in maternal health.

A family with ≤2 children indicates minimal experience with pregnancy; therefore, the husband is motivated to participate in pregnancy care and expected to play an active role in maintaining the wife's health during pregnancy. This result is consistent with those of studies reporting that husbands who do not provide SIAGA support result in multigravidas [26]. Men who have two children are more likely to be involved in reproductive health services [19]. These findings support the family planning (KB) programs related to the “2 children was better” policy; therefore, men can be more involved and participate in improving maternal health, especially ANC.

The economic aspect is an important factor hindering male participation in the care of pregnant women because lack of money limits the involvement of men in ANC [11, 16, 27]. Men employed in low-income work find allocating time to participate in pregnancy care challenging [23]. Low incomes lead to lowering the family's involvement in the health of pregnant women due to the high cost of living, especially smoking, which is prioritized more than the health of the pregnant mother [28].

Men with a good knowledge of pregnancy participate more in ANC [11], whereas men who do not are under the misconception that pregnancy care is the duty of the wife. This misconception is a gender bias that prevents men from taking an active role in the health care of their wives. Men with poor knowledge will misconceive that pregnancy care is the wife's duty. This mindset is a part of the gender bias that prevents men from taking an active role in the health care of their wives [25].

Knowledge influences male participation in ANC because it has great potential in overcoming the misconceptions and myths that hinder male participation in maternal care [16]. Men with higher levels of education tend to be more involved in reproductive health care. This is related to higher income levels and greater access to the media, which provides information about reproductive health [19]. The low knowledge barrier to male participation in ANC includes negative public perceptions of men who play an active role in maintaining the health of their families, a lack of knowledge about men's role in maternity care, and a poorly designed/implemented health-care system to facilitate male inclusion.

### 4.1. Study Limitation

In this study, we used median values to transform nonnormal distribution of continuous variables into categorical variables with high or low male participation in ANC. Although this transformed data analysis and result presentation, the use of artificial categorical variables could influence results [29]. However, in this study, the resulted cutoff is not preformed widely as measurement nor diagnostic artificial/sophisticated clinical tools. Additionally, we have used the gold standard of measurement to minimize distortion of results.

## 5. Conclusion

Most respondents reported low participation in ANC, especially regarding accompanying their wives for pregnancy testing, accompanying their wives during pregnancy check-ups, helping with housework when their wives were pregnant, reminding their wives to exercise during pregnancy, and arranging blood donors in anticipation of pregnancy-related emergencies. Factors related to the level of male participation in ANC included age, number of children, income, and knowledge. Knowledge was found to be the main factor associated with male participation in ANC in Muaro Jambi Regency. Therefore, health promotion programs are needed to empower men to participate in ANC by providing communication, education, and information.

## Figures and Tables

**Table 1:** Respondent characteristics and background.

Variable	Category	Frequency	(%)
Age (years)	(i) 21–24	7	(1.8)
	(ii) 25–34	124	(32.5)
	(iii) 35–44	174	(45.7)
	(iv) ≥45	76	(20.0)
First age of marriage	(i) 16–20	67	(17.6)
	(ii) 21–30	285	(74.8)
	(iii) ≥31	29	(7.6)
Number of children	(i) ≤2	295	(77.4)
	(ii) 3–5	85	(22.3)
	(iii) 6	1	(0.3)
Value of children (the number of willing children)	(i) ≤2	226	(59.3)
	(ii) 3-5	152	(39.9)
	(iii) 6	3	(0.8)
Income (IDR∗)	(i) ≤2,500,000	263	(69.0)
	(ii) 2,600,000–5,000,000	112	(29.4)
	(iii) 5,100,000–10,000,000	6	(1.6)
Husband's education	(i) Not attended school	1	(0.3)
	(ii) Primary school	112	(29.4)
	(iii) Junior high school	115	(30.2)
	(iv) Senior high school	139	(36.5)
	(v) University	14	(3.7)
Wife education	(i) Primary school	96	(25.2)
	(ii) Junior high school	141	(37.0)
	(iii) Senior high school	134	(35.2)
	(iv) University	10	(2.6)
Knowledge	(i) Low	165	(43.3)
	(ii) Good	216	(56.7)
Distance	(i) Far	36	(9.4)
	(ii) Near	345	(90.6)
Cost	(i) Expensive	122	(32.0)
	(ii) Cheap	259	(68.0)
Transportation	(i) Difficult	27	(7.1)
	(ii) Easy	354	(92.9)
Health insurance	(i) None	120	(31.5)
	(ii) Have	261	(68.5)
Service quality	(i) Poor	180	(47.2)
	(ii) Good	261	(52.8)
Attitude of officers	(i) Less friendly	96	(25.2)
	(ii) Friendly	285	(74.8)
Communication	(i) Poor	288	(75.6)
	(ii) Good	93	(24.4)
Social media information	(i) No	273	(71.7)
	(ii) Yes	108	(28.3)
Culture	(i) Less supportive	112	(29.4)
	(ii) Supportive	269	(70.6)

IDR: Indonesian Rupiah.

**Table 2:** Level of male participation in antenatal care.

Indicator	Frequency	(%)
(1) Plan the time and place for ANC with the wife	253	(66.4)
(2) Take the wife for pregnancy test	138	(36.2)
(3) Accompany the wife during pregnancy examinations	136	(35.7)
(4) Discuss wife's pregnancy with health workers	218	(57.2)
(5) Help pregnant wife with household chores	135	(35.4)
(6) Remind the wife to exercise during pregnancy as recommended	153	(40.2)
(7) Not smoke near pregnant wife	232	(60.9)
(8) Prepare potential blood donors for emergencies during pregnancy	109	(28.6)
Level of male participation in ANC:		
(i) Low participation	224	(58.8)
(ii) High participation	157	(41.2)

ANC: antenatal care.

**Table 3:** Relation of respondent characteristics and background with male participation in antenatal care.

Variable	Low participation		High participation		*X* ^2^	*pvalue*
	*n*	%	*n*	%		
Age						
(i) ≤30 years old	64	70.3	27	29.7	6.568	0.010∗∗
(ii) >30 years old	160	55.2	130	44.8		
Age of first marriage						
(i) <25 years old	113	60.4	74	39.6	0.405	0.524
(ii) ≥25 years old	111	57.2	83	42.8		
Number of children						
(i) >2	56	65.9	29	34.1	2.270	0.132
(ii) ≤2	168	56.8	128	43.2		
Value of children						
(i) ≤2 children	148	65.2	79	34.8	9.512	0.002∗∗
(ii) >2 children	76	49.4	78	50.6		
Income						
(i) Low	164	62.4	99	37.6	4.454	0.035∗
(ii) High	60	50.8	58	49.2		
Husband's education						
(i) Low	146	64.0	82	36.0	6.441	0.011∗
(ii) High	78	51.0	75	49.0		
Wife's education						
(i) Low	150	63.3	87	36.7	5.238	0.022∗
(ii) High	74	51.4	70	48.6		
Knowledge						
(i) Not good	126	76.4	39	23.6	37.089	0.001∗∗
(ii) Good	98	45.4	118	54.6		
Distance						
(i) Far	17	47.2	19	52.8	2.197	0.138
(ii) Near	207	60.0	138	40.0		
Cost						
(i) Expensive	66	54.1	56	45.9	1.632	0.201
(ii) Cheap	158	61.0	101	39.0		
Transportation						
(i) Difficult	16	59.3	11	40.7	0.003	0.959
(ii) Easy	208	58.8	146	41.2		
Health insurance						
(i) No.	70	58.3	50	41.7	0.015	0.902
(ii) Yes	154	59.0	107	41.0		
Service quality						
(i) Poor	102	56.7	78	43.3	0.574	0.449
(ii) Good	121	60.5	79	39.5		
Attitude						
(i) Less friendly	57	59.4	39	40.6	0.018	0.893
(ii) Friendly	167	58.6	118	41.4		
Communication						
(i) Poor	184	63.9	104	36.1	12.648	0.001∗∗
(ii) Good	40	43.0	53	57.0		
Social media information						
(i) No.	175	64.1	98	35.9	11.208	0.001∗∗
(ii) Yes	49	45.4	59	54.6		
Culture						
(i) Less supportive	75	67.0	37	33.0	4.372	0.037∗
(ii) Supportive	149	55.4	120	44.6		

Note: ∗*p* ≤ 0.05; ∗∗*p* ≤ 0.01.

**Table 4:** Multivariate logistic regression analysis: associated factors of male participation in antenatal care.

Variable	*B*	P-W*ald*	OR Exp(B)	95% CI
Age	0.620	0.029∗	1.858	1.066–3.240
Number of children	1.068	0.001∗∗	2.909	1.532–5.522
Income	0.540	0.028∗	1.715	1.060–2.775
Knowledge	1.310	0.001∗∗	3.706	2.320–5.919

Note: ∗*p* ≤ 0.05; ∗∗*p* ≤ 0.01.

## Data Availability

The datasets generated and/or analyzed during the current study are not publicly available due to confidentiality, but are available on reasonable request.
